# Heads or Tails: Genotyping of Hepatitis C Virus Concerning the 2k/1b Circulating Recombinant Form

**DOI:** 10.3390/ijms17091384

**Published:** 2016-08-23

**Authors:** Wim Schuermans, Hans Orlent, Isabelle Desombere, Patrick Descheemaeker, Hans Van Vlierberghe, Anja Geerts, Xavier Verhelst, Marijke Reynders, Elizaveta Padalko

**Affiliations:** 1 Department of Clinical Chemistry, Microbiology and Immunology, Ghent University and Hospital, Ghent 9000, Belgium; Wim.Schuermans@ugent.be (W.S.); Elizaveta.Padalko@uzgent.be (E.P.); 2 Department of Gastroenterology and Hepatology, AZ Sint-Jan Bruges-Ostend, Bruges 8000, Belgium; Hans.Orlent@azsintjan.be; 3 Center for Vaccinology, Ghent University and Hospital, Ghent 9000, Belgium; Isabelle.Desombere@UGent.be; 4 Department of Laboratory Medicine, Clinical Microbiology, AZ Sint-Jan Bruges-Ostend, Bruges 8000, Belgium; Patrick.Descheemaeker@azsintjan.be; 5 Department of Gastroenterology and Hepatology, Ghent University and Hospital, Ghent 8000, Belgium; Hans.VanVlierberghe@uzgent.be (H.V.V.); Anja.Geerts@ugent.be (A.G.); Xavier.Verhelst@uzgent.be (X.V.); 6 School of Life Sciences, Hasselt University, Diepenbeek 3590, Belgium

**Keywords:** HCV, genotype, sequencing, misclassification, CRF 2k/1b

## Abstract

As different hepatitis C virus (HCV) genotypes respond differently to initiated therapy, correct HCV genotyping is essential. A potential risk for misclassification of the intergenotypic HCV circulating recombinant form (CRF) 2k/1b strains exists, depending on the genotyping method used. The aim was to investigate the differences in HCV genotyping methods with regard to CRF 2k/1b and to gain insight in the prevalence of the CRF 2k/1b. Genotyping results by Versant HCV Genotype Assay were compared with nonstructural protein 5B (NS5B) sequencing. In total, from November 2001 until March 2015, 3296 serum samples were analyzed by Versant HCV Genotype Assay. As misclassified CRF is harbored among HCV genotype 2, we further focused our search on 142 (4.3%) samples positive for HCV genotype 2. On 116 (81.7%) retrieved samples, the NS5B sequencing was performed. Twelve out of the 116 retrieved samples (10.3%) were classified as CRF 2k/1b by sequencing of the NS5B region. Ten of these 12 samples were originally misclassified as genotype 2a or 2c, while 2 of them were misclassified as genotype 2. Our results show that the current prevalence of CRF 2k/1b is underestimated. The importance of correct HCV genotyping is emphasized, considering the tailored choice of treatment regimen and overall prognosis.

## 1. Introduction

Globally, around 140 million people live with chronic hepatitis C virus (HCV) infection. On the other hand, about 15% to 45% of initially-infected persons clear the virus within six months of infection without any treatment. Chronic HCV infection can cause cirrhotic development of the liver and evolution to hepatocellular carcinoma. The risk of cirrhosis is estimated at 15% to 30% within 20 years [1]. Obviously, the target of antiviral therapy is to prevent this disease progression. Correct HCV genotyping, aside from the level of viremia, is fundamental to solid decision-making concerning the type and duration of antiviral therapy [1,2,3].

HCV was identified by molecular means in 1989 by screening infected chimpanzee blood for viral RNA [4]. HCV is characterized by a high degree of genetic heterogeneity, on which its classification is based [5]. HCV strains isolated in different parts of the world were classified into six major genotypes (i.e., genotypes one to six) and numerous subtypes. The HCV genomic RNA consists of three distinct regions: a 5′ untranslated region (UTR), a long open reading frame (ORF) and a short 3′ UTR. The 5′ UTR is the most conserved one in the genome, and hence is used as a target for HCV genotyping [6].

The structural organization of the ORF genome-encoded protein is as follows: NH_2_–C–E1–E2–p7–NS2–NS3–NS4A–NS4B–NS5A–NS5B–COOH. This polyprotein yields at least ten different parts through the cleaving activity of host and viral proteases. The harvest includes three structural proteins (core, E1, and E2), a small protein (p7), and six non-structural proteins (NS2, NS3, NS4A, NS4B, NS5A and NS5B). The core protein forms the viral capsid through its RNA-binding capacity. The two envelope glycoproteins (E1 and E2) make viral entry possible. This constitutes a crucial step in its viral life cycle. E2 contains highly variable regions (HVRs) with amino acid sequences differing up to 80% between different isolates. p7, an integral membrane protein, is a member of the viroporin family, and could act as an ion channel with an essential role in virus infectivity. The C-terminus of NS2 and the N-terminus of NS3 constitute the NS2/3 protease. This protease cleaves the site between NS2 and NS3, as its name suggests. The C-terminal domain of NS3, on the other hand, has helicase/NTPase activity. Its functions include RNA binding and unwinding of regions of extensive secondary structure, aside from RNA-stimulated NTPase activity. NS4A acts as a cofactor for NS3 protease activity. The NS3/4A protease activity results in cleavage of the viral nonstructural proteins. Recruitment of other viral non-structural proteins to form the replication complex is the function of NS4B. The role of NS5A is not entirely clear. It is also probably important in viral replication. NS5B—being an RNA-dependent RNA polymerase (RdRP)—ensures the synthesis of new genomic RNA, and is consequently considered a central component of the HCV replication complex [7,8]. The high genomic heterogeneity of HCV is the result of the lack of proof-reading activity of this RdRP [9].

The discovery of a viable intergenotypic circulating recombinant form (CRF) in St. Petersburg suggests that recombination can also lead to genetic diversity in HCV [10]. The concerned recombinant form has a 5′ genome region that is most closely related to genetic subtype 2k, and a 3′ genome region that is most closely related to the global epidemic genetic subtype 1b, and hence is designated RF1_2k/1b. Sequencing of the E2–p7–NS2 region mapped the crossover point within the NS2 region, between nucleotides 3175 and 3176. The presence of 1b in this CRF is quite logical, since it is one of the most prevalent subtypes worldwide [11]. Subtype 2k probably arrived in the former Soviet Union from West Africa through the historical trans-Atlantic slave trade [12]. By using a Bayesian phylogenetic approach, the time of origin of this particular recombination event is estimated between 1923 and 1956 [13]. Interestingly, a nationwide network of blood transfusion centers throughout the Soviet republics was established during this period [14]. Obviously, these centers proved to be useful during the Second World War, and have most likely played a significant role in HCV transmission. The breakup of the former Soviet Union subsequently led to a dramatic increase in intravenous drug use, still a major risk behavior for HCV infection [15]. However, transmission has slowed with the onset of anti-HCV screening of blood donors since the early 1990s. Since the first description of RF1_2k/1b in intravenous drug users in St. Petersburg, it has been isolated throughout Eurasia [16,17,18,19,20].

There is a risk of misclassification of this recombinant form, depending on the genotyping method used [21,22]. This has important therapeutic and prognostic consequences. Ribavirin (RBV) should be given with a combination of at least two direct acting antivirals (DAAs) in the case of the CRF 2k/1b. A significant amount of relapses will occur in this scenario when therapy is restricted to RBV with a single DAA [23]. Because of these therapeutic and prognostic consequences of HCV genotyping, a retrospective analysis of HCV-positive serum samples was performed using two different methods. This approach would also deliver useful information on the prevalence of the CRF in our patient population that has not been assessed up to now.

## 2. Results

From November 2001 until March 2015, 3296 serum samples were examined for HCV genotype determination at the Ghent University Hospital, using the Versant HCV Genotype 2.0 Assay (Tarrytown, NY, USA). Before November 2008, the Versant HCV Genotype 1.0 Assay was utilized.

These two versions of the Versant HCV Genotype Assay are comparable for the distinction of genotype 2 [24].

One hundred and forty-two samples (or 4.3%) proved positive for HCV genotype 2, which is concordant with the prevalence in literature [25].

We were able to retrieve 116 (81.7%) of these samples. To check for correlation between the Versant HCV Genotype Assay and the sequencing method, they were subsequently sent to the Bruges Sint-Jan hospital (Bruges, Belgium) for NS5B sequencing. Put otherwise, the “tail” region of the HCV RNA genome was sequenced, while this information was already available for the “head” region.

For three of these samples, insufficient volume was available for a required rerun. One sample did not yield an amplicon. This sample had a low viral load (i.e., <6 × 10^4^ IU/mL).

**Figure 1:**
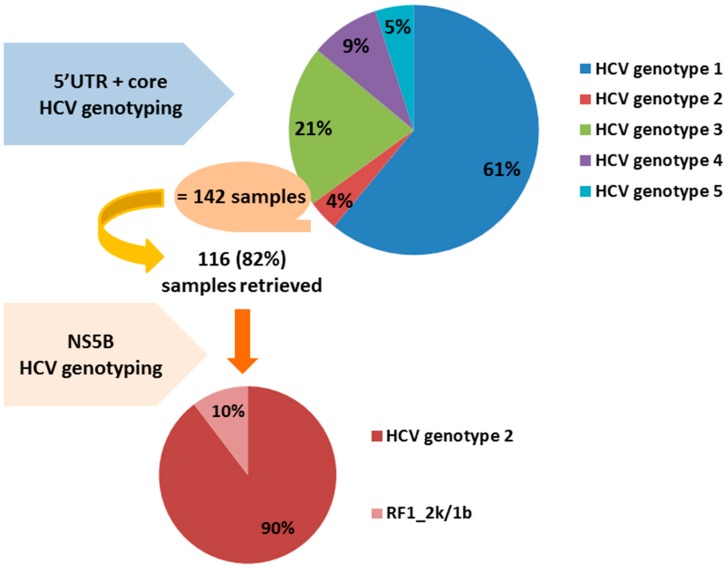
Summary of the results obtained: 12 out of the 116 HCV genotype 2 samples retrieved or 10% were classified eventually as RF1_2k/1b by sequencing of the NS5B region. RF1_2k/1b: recombinant form 2k/1b; HCV: hepatitis C virus; UTR: untranslated region; NS5B: nonstructural protein 5B.

Twelve out of the 116 retrieved samples (10.3%) were classified as RF1_2k/1b by sequencing of the NS5B region. Ten of these 12 samples were initially misclassified as genotype 2a or 2c, while two of them were misclassified as genotype 2 by the Versant HCV Genotype 2.0 Assay. Figure 1 provides the graphic summary of the results obtained. It is known that the Versant HCV Genotype 2.0 Assay does not discriminate well between subtypes of genotype 2 [20,22].

## 3. Discussion

The Versant HCV Genotype 1.0 Assay was utilized before November 2008, as already mentioned. The improvement of the second generation assay—including the core region probes—lies mainly in the distinction between subtype a and subtype b of genotype one. Obviously, the version of the Versant HCV Genotype Assay has no impact on the distinction of genotype two [24].

Because of possible therapeutic implications, it would be valuable to genotype at least two different genomic regions, when HCV genotype two resulted from the Versant HCV Genotype 2.0 Assay [26].

According to the quality control for molecular diagnostics (QCMD) results, participant performance indicated that the 5′ UTR target was less able to subtype HCV 1a and 1b than NS5B (confer report QAV034117 HCVGT13). When HCV genotype two is found with the Versant HCV Genotype 2.0 Assay targeting the “head” regions of HCV, NS5B sequencing targeting the “tail” region should be performed in order to be able to detect CRF 2k/1b. In other words, sequencing the “tail” region in HCV genotyping will suffice to unmask RF1_2k/1b. As mentioned, the recombinant virus 2k/1b arose somewhere between 1923 and 1956, and these strains underwent their own genomic evolution (also at the level of NS5B), identifiable as a clade within HCV 1b when using NS5B sequence analysis. Consequently, NS5B sequencing analysis is accurate and discriminative enough to confidently identify HCV 2k/1b strains.

Our results clearly show that the current prevalence of RF1_2k/1b is underestimated and has not been systematically addressed up to now, including European HCV patients with a history of injection drug use [27]. We would like to emphasize the importance of correct HCV genotyping, considering upon tailored choice of treatment regimen and overall prognosis. The mentioned DAAs reflect quite recent advances in molecular biology. Our RF1_2k/1b-positive cohort population was either lost to follow-up or treated with classic state-of-the-art therapy, due to the historic aspect of the studied population. Gathering information about the transmission route, geographic origin, and clinical follow-up of the RF1_2k/1b positive cohort appeared to be very challenging and fragmentized, reflecting sometimes very difficult settings of the patients—e.g., penitentiary residence with complete lack of information on these issues. Due to the incomplete data and difficulties that we faced trying to collect them, it is recommended that this information be acquired in a prospective manner rather than in the current retrospective design of the study with data going back to 2001.

Regarding susceptibility of RF1_2k/1b to treatment, discordant results can be found in the literature: some claim higher susceptibility to pegylated interferon (pegINF)/RBV [28] in contrast to others [29]. DAAs in combination with RBV appear to have good activity against HCV intergenotypic recombinant strains [30]. In the case of genotype two, sofosbuvir in combination with RBV is the therapy of choice. Since genotype one is included in the tail of RF1_2k/1b, RBV should be given with a second DAA in addition to sofosbuvir. Relapse rates of more than 90% have been described when therapy is restricted to RBV with sofosbuvir as the only DAA [23]. The sting is in the tail, indeed. However, even in the latest published guidelines, no clear recommendations exist on duration or optimal therapy for CRF [31].

Further studies in this field are required in order to fully establish the actual epidemiological burden and clinical impact of HCV RF1_2k/1b.

## 4. Materials and Methods

### 4.1. Serum Samples

Samples submitted for HCV genotyping from November 2001 until March 2015 were included in the study. During this period of 161 months, 3296 serum samples were analyzed at the Ghent University Hospital, using the Versant HCV Genotype 2.0 assay (LiPA; Siemens Healthcare, Erlangen, Tarrytown, NY, USA). The Versant HCV Genotype 1.0 assay was utilized before November 2008. The yield for HCV genotype 2 was 142 samples (4.3%). One hundred and sixteen (81.7%) retrospectively retrieved samples, representing 114 patients, were subsequently sent to the Bruges Sint-Jan hospital for NS5B sequencing. Both methods used in this study—Versant HCV Genotype 2.0 Assay at Ghent University Hospital and NS5B sequencing at Bruges Sint-Jan Hospital—fully fulfill quality assurance obligations required in Belgium (i.e., BELAC-accreditation meaning ISO-accreditation) involving obligatory participation and succeeding in External Quality Assessment Programs. Ethical approval was obtained from the ethics committee of the Ghent University Hospital, Bruges, Belgium, with registration number B67021525240.

### 4.2. RNA Extraction and cDNA Synthesis and Amplification

RNA was extracted using the NucliSens EasyMAG™ (bioMérieux, Marcy l’Etoile, France). An input volume of 110 µL of serum is required, which results in an equal elution volume.

The Versant HCV Amplification Kit (LiPA; Siemens Healthcare, Erlangen, Germany) was utilized for reverse transcription (RT) and requires 20 µL of RNA extract. Subsequent amplification through PCR of the 5′ UTR and core region of the HCV genome was performed with the GeneAmp PCR System 9600 (Applied Biosystems, Foster City, CA, USA). All tests were performed according to the manufacturer’s instructions.

### 4.3. Reverse Hybridization

At the Ghent University Hospital, the Versant HCV Genotype 1.0 assay (LiPA; Siemens Healthcare, Erlangen, Germany) was used until November 2008. Starting from this date, the Versant HCV Genotype 2.0 Assay has been utilized. Ten microliters of PCR product is required for this second-generation line probe assay, utilizing reverse hybridization. RT-PCR amplification yield of the 5′ UTR and core region of the HCV genome is biotinylated and hybridized to immobilized oligonucleotide probes. These probes are specific for the above-mentioned regions of different HCV genotypes. Subsequently, conjugate—i.e., alkaline phosphatase-labeled streptavidin—is bound to the biotinylated hybrid. When the substrate (5-bromo-4-chloro-3-indolyl phosphate p-toluidine salt/nitroblue tetrazolium chloride (BCIP/NBT) chromogen) comes into play, a purple/brown precipitate may result as the substrate reacts with the formed streptavidin–alkaline phosphatase complex. The Versant strips have three control lines and 22 parallel DNA probe lines containing sequences specific for HCV genotypes one to six. The conjugate control line monitors the mentioned color development. The amplification control at lines two and 23 hybridize the PCR products from the 5′ UTR and core regions, respectively. A specific banding pattern on the strip thus becomes visible. The specific HCV genotype is determined by aligning the assay strips with the Versant HCV Genotype 2.0 Assay Reading Card and comparing the line patterns from the assay strips with the patterns shown on the Versant HCV Genotype 2.0 Assay Interpretation Chart.

### 4.4. DNA Sequencing and Sequence Analysis

The NS5B sequencing method described by Murphy et al. [32] is the method of choice at the Bruges Sint-Jan Hospital. First, 389 BPs of the *NS5B* gene are amplified through one step RT-PCR. This amplicon is subsequently purified, using two enzymes, Exonuclease I and Shrimp Alkalic Phosphatase. Exonuclease I hydrolyzes single stranded DNA in a 5′ to 3′ direction, degrading the primers. Alkalic Phosphatase inactivates the dNTPs by dephosphorylation. Secondly, cycle sequencing according to the method of Sanger is performed [33]. Amplicons are sequenced in both directions using the sense and antisense amplification primers. The yield is purified with the aid of Sephadex gel filtration in the third step. Finally, the DNA fragments are separated from each other through capillary electrophoresis with the laser at the end, exciting the final fluorescent base. This eventually leads to recognition of the particular NS5B nucleotide sequence. The HCV genotype is determined by comparing this NS5B nucleotide sequence with known NS5B sequences in available databases. The National Center for Biotechnology Information, Basic Local Alignment Search Tool (NCBI BLAST) is used for this purpose.

## Supplementary Materials

Supplementary materials can be found at www.mdpi.com/1422-0067/17/9/1384/s1.
